# 

**Published:** 1924-05

**Authors:** 


### HOSPITAL and HEALTH

REVIEW

*Established 1886* *No.* 1874. *Vol. LXXIII*

*New Series.* Vol. III. No. 32. *May,* 1924. Price Sixpence

CONTENTS.

| | PAGE |
|---|---|
| Closing the Gap | 127 |
| The Worker and his Hour of Need | 128 |
| Notes and Comments :— The British Empire Exhibition—More Housing Schemes—Standardising Summer Time—The Part-Time Medical Officer—Influenza and Infant Mortality—The Dogs Bill—Pigments in Animal Life—"The Staff of Life"—Starving the Nurse—Venereal Disease Campaign in Germany—Drunk for Threepence — Maternity Mortality — A "Cleanliness Week"—The Prevention of Congenital Syphilis | 129 |
| Hospital Men of Mark : Mr. Frederick Acton and Mr. Peter McColl | 133 |
| Correspondence | 134 |
| Sunday Afternoon with the Wounded. By Theodore Ruete | 135 |
| National Health Insurance Notes | 136 |
| Death Rate in Childhood. By Sir Arthur Newsholme, K.C.B. | 137 |
| Hospitals and Income Tax. By Joseph E. Stone, F.S.A.A., F.S.S. | 139 |
| The Queen at the New Nurses' Hostel, Windsor | 140 |
| Hospital Progress and Finance | 141 |
| Public Health : Interviews with Local Authorities : Cardiff | 143 |
| Our Health Week Competition : The Winning Papers | 146 |
| The Hospital and Nursing Exhibition | 150 |
| The Battle for Infant Life | 151 |
| Reviews of Books | 152 |
| Hospital and Health News | 155 |
| Echoes of the Month | 157 |
| The Hospital and Nursing Exhibition : Some of the Stalls | 158 |
| Public Health in Parliament | 162 |
| Recent Appointments | 162 |
| Answers to Correspondents | 162 |

